# Periprosthetic bone density changes after MiniHip^TM^ cementless femoral short stem: one-year results of dual-energy X-ray absorptiometry study

**DOI:** 10.1051/sicotj/2016033

**Published:** 2016-11-18

**Authors:** Ahmet Ercan, Sherif M. Sokkar, Gebhard Schmid, Timm J. Filler, Ashraf Abdelkafy, Joerg Jerosch

**Affiliations:** 1 Department of Orthopedics, Trauma Surgery and Sports Medicine, Johanna Etienne Hospital AM Hasenberg 46 41462 Neuss Germany; 2 Orthopaedic Surgery and Traumatology Department, Suez Canal University Circular Road 41522 Ismailia Egypt; 3 Department of Diagnostic Radiology, Johanna Etienne Hospital AM Hasenberg 46 41462 Neuss Germany; 4 Department of Anatomy, Heinrich-Heine University of Duesseldorf, Universitaetsstrasse 1 40225 Duesseldorf Germany

**Keywords:** Periprosthetic bone density, MiniHip, Cementless, Short stem, Dual-energy X-ray absorptiometry

## Abstract

*Introduction*: The purpose of the current study was to investigate the reaction of the femur to the implantation of the MiniHip^TM^ in terms of: (1) bone density change during one year; (2) correlations between stem length, CCD (caput-collum-diaphyseal), femoral offset, T-value, and bone density; (3) other co-variables that influence the change of bone density.

*Patients and methods*: MiniHip^TM^ implant was performed for 62 patients. The age range of the patients who underwent treatment was 25–78 years. Periprothestic bone density was determined within two weeks postoperatively, after three, six, and twelve months utilizing the DEXA scan.

*Results*: The highest change was observed in the first three months post-implantation, while significant decrease in density was recorded at proximal Gruen zones 1, 2, and 7, and at distal Gruen zone 4. The decrease in density reached a plateau between the third and sixth months after operation. Afterwards, bone density recovered up to the 12th postoperative month. The correlation analysis showed significant difference between Gruen zone 1 and stem size and CCD. The same significant trend was not reached for Gruen zone 7. Femoral offset showed no correlation. Covariance analysis was unable to establish connection of the results with diagnosis, pairings, or gender.

*Discussion*: MiniHip^TM^ densitometric results are promising and comparable to good results of the other representatives of the femoral neck partially-sustaining short stem prostheses with a lower proximal bone density reduction. Periprosthetic bone resorption is a multifactorial process where stem size, CCD angle, and patient-specific variables such as T-value have an impact on the periprosthetic bone remodeling. In particular, this applies to Gruen zone 1.

## Introduction

Cementless femoral stem fixation has now become the method of choice in total hip replacement (THR) [1]. The early cementless stems were either straight or curved and engaged the femur in proximal metaphysis as well as distally [2]. Despite successful long-term results with most designs, stress shielding and thigh pain may occur [3].

Short cementless femoral stems, also called bone-conserving cementless stems, have been introduced to preserve the proximal bone stock and allow more physiological proximal loading [4]. The available short stems differ in designs and outcomes [5]. Short stems are categorized into four groups: Type 1 femoral neck only, Type 2 calcar loading, Type 3 lateral flare calcar loading, and Type 4 shortened tapered. In Types 1 through 4, as the number increases so does the loading across the proximal part of the femur [1]. There is still no accepted and validated definition in the literature on how long short stem prosthesis should be. Resection level classifications have been proposed, where short stem prostheses were classified into: neck-sustaining, neck-partially-sustaining, and neck-resected [6].

Short stem prostheses are increasingly in demand and in the number of implantations, especially in younger patients [6, 7]. Studies have reported good mid-term follow-up results [8]. However, still there is no consensus regarding potential benefits and limitations of these specific implant designs. Potential issues are revision rates and complications (e.g. periprosthetic fractures, subsidence, and the chronic pain in the area of the greater trochanter).

**Figure 1.**
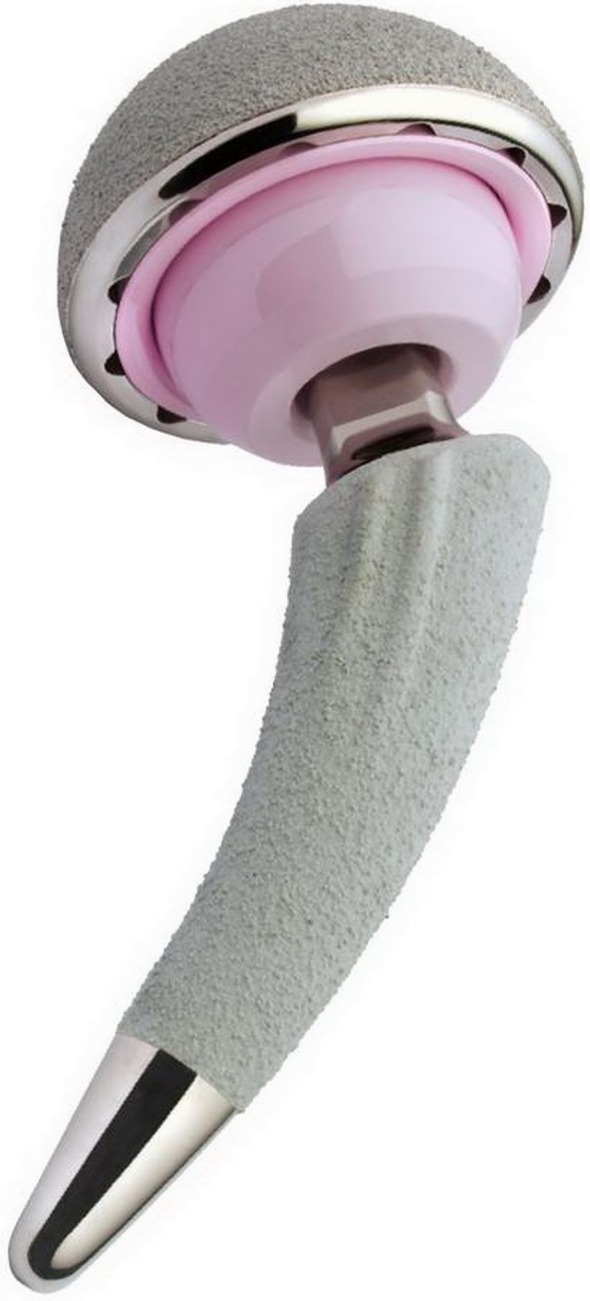
MiniHip^TM^ (Corin Group PLC, Cirencester, UK).

The short stem hip prosthesis MiniHip^TM^ (Corin Group PLC, Cirencester, UK) belongs to the family of femoral neck-sustaining short stem prostheses and was introduced in 2007 (Figure 1). Clinical follow-up studies of the MiniHip^TM^ showed good early to mid-term results [9, 10].

The purpose of the current study was to investigate the reaction of the femur to the implantation of the MiniHip^TM^ in terms of:

- Bone density change during one year after implantation of the MiniHip^TM^.
- Correlations between stem length, CCD (caput-collum-diaphyseal) angle (angle between the longitudinal axes of the femoral neck and shaft), femoral offset, T-value, and bone density change.
- Other co-variables that might influence the change of bone density.

## Patients and methods

It is a single surgeon, single center (The clinic of Orthopedics, trauma Surgery and Sport Medicine of the Johanna Etienne Hospital in Neuss, Germany), retropective consecutive case series study. Sixty-two patients who underwent THR using MiniHip^TM^ short stem prosthesis were included in the current study. The average age at surgery was 56.6 years (range 25–78). There were 34 females and 28 males. The study involved 31 right hips and 31 left hips. The average patients’ weight at the time of surgery was 83.7 kg (range 53–140), while the average body mass index (BMI) was 28.57 (range 18.29–49.6).

Inclusion criteria were: osteoarthritis, hip dysplasia, avascular necrosis, and femoral neck fractures (Table 1), while exclusion criteria were: age above 80, deformities, and post-traumatic conditions.

**Table 1.** Distribution of the indications of MiniHip^TM^ short stem prosthesis among patients.

Diagnosis	Frequency	Percentage (%)
Osteoarthritis	49	79
Hip dysplasia	7	11.3
Avascular necrosis	4	6.5
Femoral neck fracture	2	3.2
Total	62	100

The Ethics Committee of the medical school of Heinrich-Heine University, Düsseldorf, Germany approved the study under the No. 4825.

### Prosthesis features

The MiniHip^TM^ short stem prosthesis design is based on a detailed evaluation of Computed Tomography (CT) data of 200 femora and was determined based on their specific proximal femur points [11]. The MiniHip^TM^ shaft is available in nine sizes with a gradually increasing neck length. The surface consists of a Bi-Coat coating of titanium and hydroxyapatite, both of which were each mounted with a plasma spray method to the surface and have a thickness of 100 μm. The distal tip of the prosthesis is polished and is designed to prevent a fixation in this area, thus reducing the risk of an anterior thigh pain.

### Surgical technique

In all patients the so-called ALMI (anterolateral minimally invasive) approach was used [12]. The desired cup position was set at 40–50° to the horizontal plane with about 10° anteversion. In MiniHip^TM^ prosthesis, a standard resection of the femoral neck is not performed, but rather, depending on the preoperative CCD angle and thus restoration of the anatomy is guaranteed by selecting the resection plane at the femoral neck.

**Figure 2.**
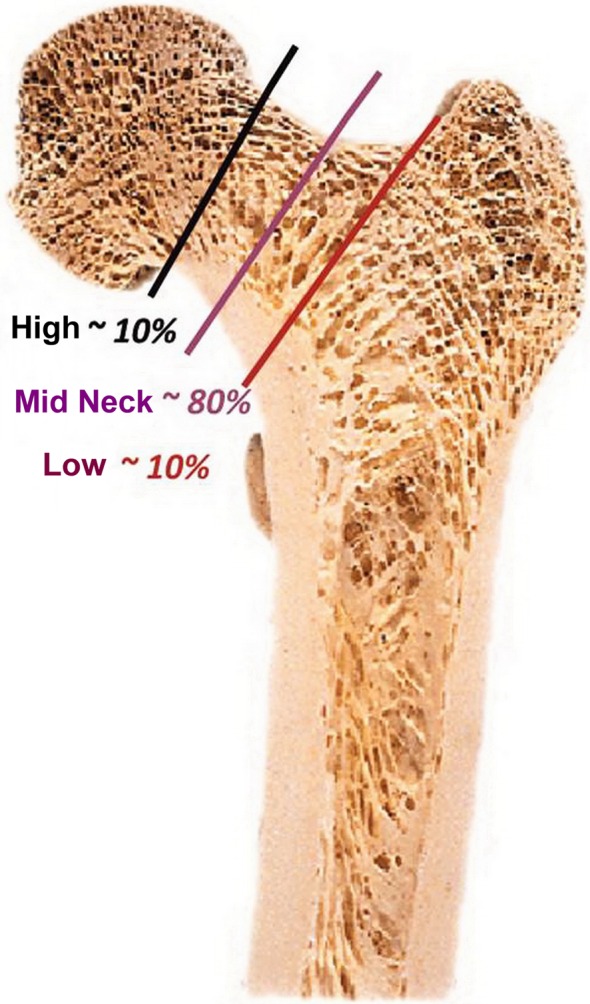
Resection levels with frequencies.

A reference point is not the greater trochanter, but the piriformis fossa. In a normal hip, resection occurs in the femoral neck parallel to the mid-plane of the head and neck level with a so-called 90–90 resection. At a valgus hip, a deep resection is performed, while, at a varus hip a direct sub-capital resection is performed in order to restore offset and CCD angle (Figure 2) [11]. After opening the femoral medullary cavity; it is not processed with rasps, but by the use of impactors, so that the spongy bone becomes compressed. After appropriate preparation, the stem is introduced.

On average, the stem size 5 was the commonest used among patients, while size one was not used in any of the patients.

Three pairings were used (Table 2):

- Ceramic-Ceramic.
- Ceramic-polyethylene (PE).
- Metal-PE.

**Table 2.** Distribution of different pairings among patients who underwent MiniHip^TM^.

Pairings	Frequency	Percentage (%)
Ceramic-ceramic	42	67.7
Ceramic-polyethylene	18	29
Metal-polyethylene	2	3.2
Total	62	100

The postoperative treatment was carried out with full weight bearing as pain tolerated with the aid of forearm crutches.

### DEXA scans

**Figure 3.**
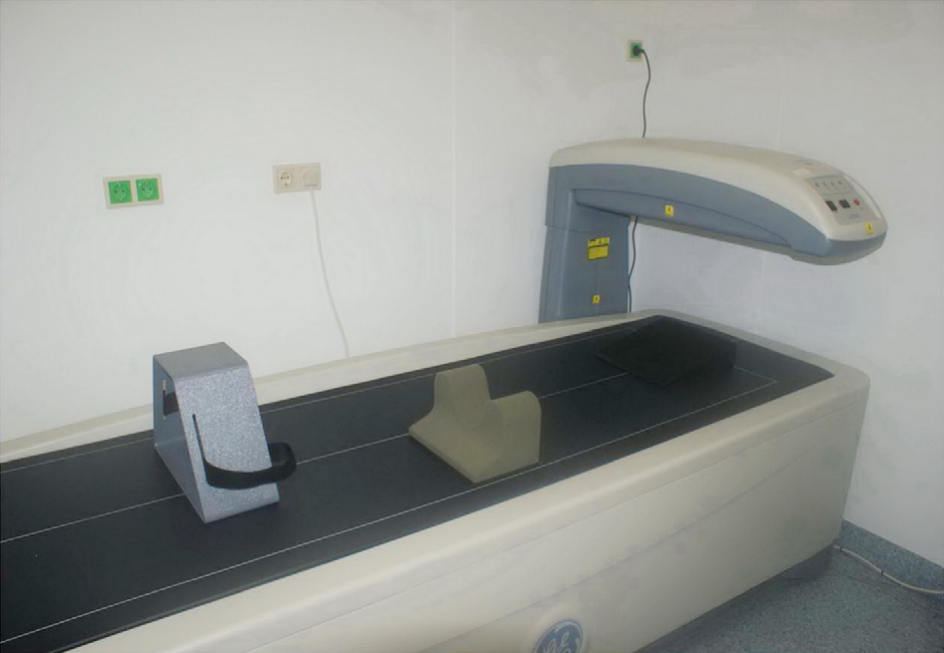
DEXA scan with the appropriate positioning aids.

The periprosthetic bone density was determined by the Dual-Energy-X-ray-Absorptiometry (DEXA) scan within the first two weeks postoperatively as basic measurement as well as three, six, and twelve months postoperatively for all patients. For this, the Lunar Prodigy^TM^ (GE Healthcare, Madison, WI) DEXA scan was used. For measurement, the patient lies on his back, in which the leg to be examined is fixed in a holder at the foot, in order to position the hip in a constant internal rotation of 5°. In addition, the knee is fixed to a foam holder (Figure 3).

A slow scan mode was utilized and the “Orthopedic Hip” software was used. The femoral stem in accordance with the zones described by Gruen et al. [13] in seven ranges “*Regions of Interest*” (ROI) subdivided in analogy to the radiographic classification. In these ranges, the metal parts have been automatically identified by the software and skipped, so that the periprosthetic bone mineral density (BMD [g/cm^2^]) could be specified precisely in the seven regions. So, the amendments of the bone structure around the prosthesis are effectively monitored.

As a control, a measurement on the lumbar spine was conducted and the T-value was determined. The radiologic analysis was performed on postoperative digitally designed standardized Beck overview recordings in the anterior-posterior beam path with the existing tools of image viewing program JiveX^®^. The femoral offsets as well as the projected CCD angle using the Lecerf method were determined [14].

All data were analyzed using the Statistical Package for the Social Sciences (SPSS) version 22.0 for Windows. Mean differences in the various series of measurements were analyzed using a repeated-measures analysis of variance (ANOVA).

Frequency distributions were analyzed with the χ^2^-test. Results with *p* < 0.05 were considered statistically significant. The significance tests were performed two-sided and carried out at non-directional hypotheses. In the case of a significant variance analysis and in order to determine which groups differed from each other, the conservative Bonferroni test was used as post hoc test of significance.

Relationships between interval scaled data were checked by calculating Pearson’s product-moment correlations and inferential statistics covered by the corresponding *t*-test for correlation coefficient. Missing values due to no-show, rejection of a measure were initially analyzed. To avoid distortion, missing values were replaced by the statistical method of multiple imputation. Five imputations were used, so that five new records were created, which were then later merged by the pooling back into a statistic.

## Results

A decrease in bone density after three months in all Gruen zones was detected (Table 3). Comparing the postoperative DEXA values (baseline) with those at three, six, and twelve months, results showed a drop in Gruen zones 1, 2, 4, and 7, respectively, which were statistically significant (Table 4). After twelve months, the changes in the previously mentioned Gruen zones remained significant, when compared to the baseline. Comparing the DEXA values at three months with those at six months showed significant increase in bone density, detected in Gruen zone 3. The same occurred when comparing DEXA values at six months with those at twelve months.

**Table 3.** Percentage of bone density changes at three, six, and twelve months postoperatively.

Gruen zones	1 (%)	2 (%)	3 (%)	4 (%)	5 (%)	6 (%)	7 (%)
Three months	−10.05	−12.37	−3.97	−6.90	−2.37	−0.81	−10.05
Six months	−12.69	−13.20	−0.86	−8.30	−3.16	+2.54	−13.87
Twelve months	−8.37	−14.59	+0.82	−6.69	−3.03	−0.41	−11.48

**Table 4.** Comparison between postoperative DEXA values and those at three, six, and twelve months and comparing values at three, six, and twelve months with each other.

Gruen zone (*p* < 0.05)	1	2	3	4	5	6	7
Postoperative vs. three months	+	+	−	+	−	−	+
Postoperative vs. six months	+	+	−	+	−	−	+
Postoperative vs. twelve months	+	+	−	+	−	−	+
Three months vs. six months	−	−	+	−	−	−	−
Three months vs. twelve months	−	−	+	−	−	−	−
Six months vs. twelve months	−	−	−	−	−	−	−

+ = Significance; − = Not significant.

**Figure 4.**
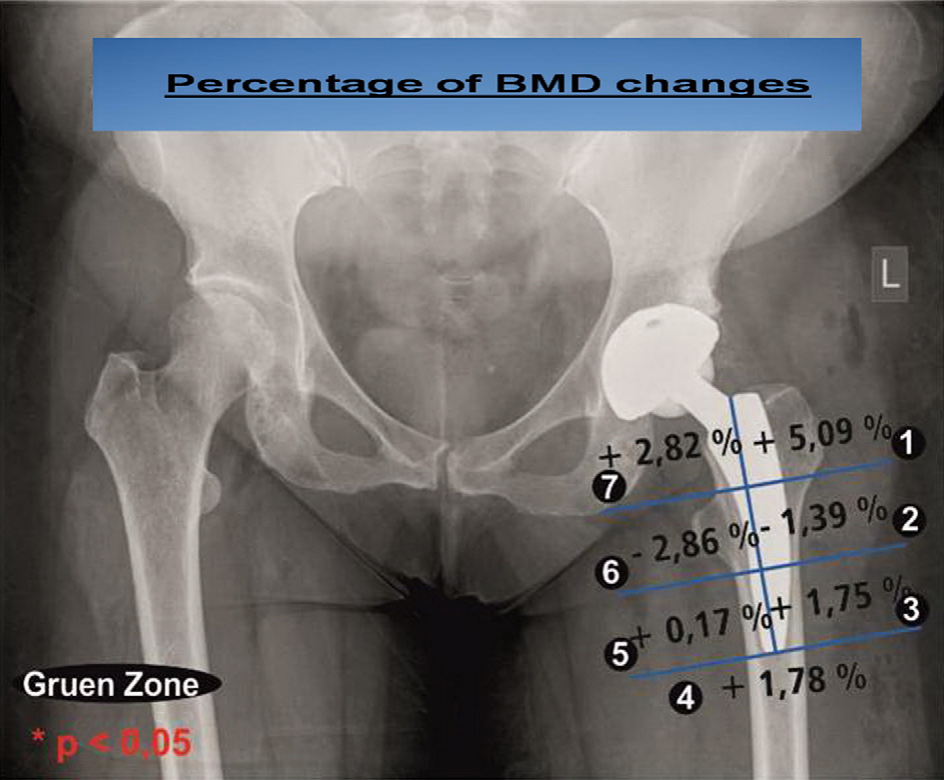
Periprosthetic bone density change six versus twelve months postoperatively.

Comparing bone density after six months with the bone density after twelve months (Figure 4), a recovery trend was recognizable in some areas. An increase in bone density was recorded both proximally in the Gruen zones 1 and 7 and distally in zones 3–5. Only in the corresponding Gruen zones 2 and 6, a slight decrease was seen. All these changes were not statistically significant.

Comparative measurements with the lumbar spine and despite the decrease in bone density, no statistically significant differences were obtained.

**Figure 5.**
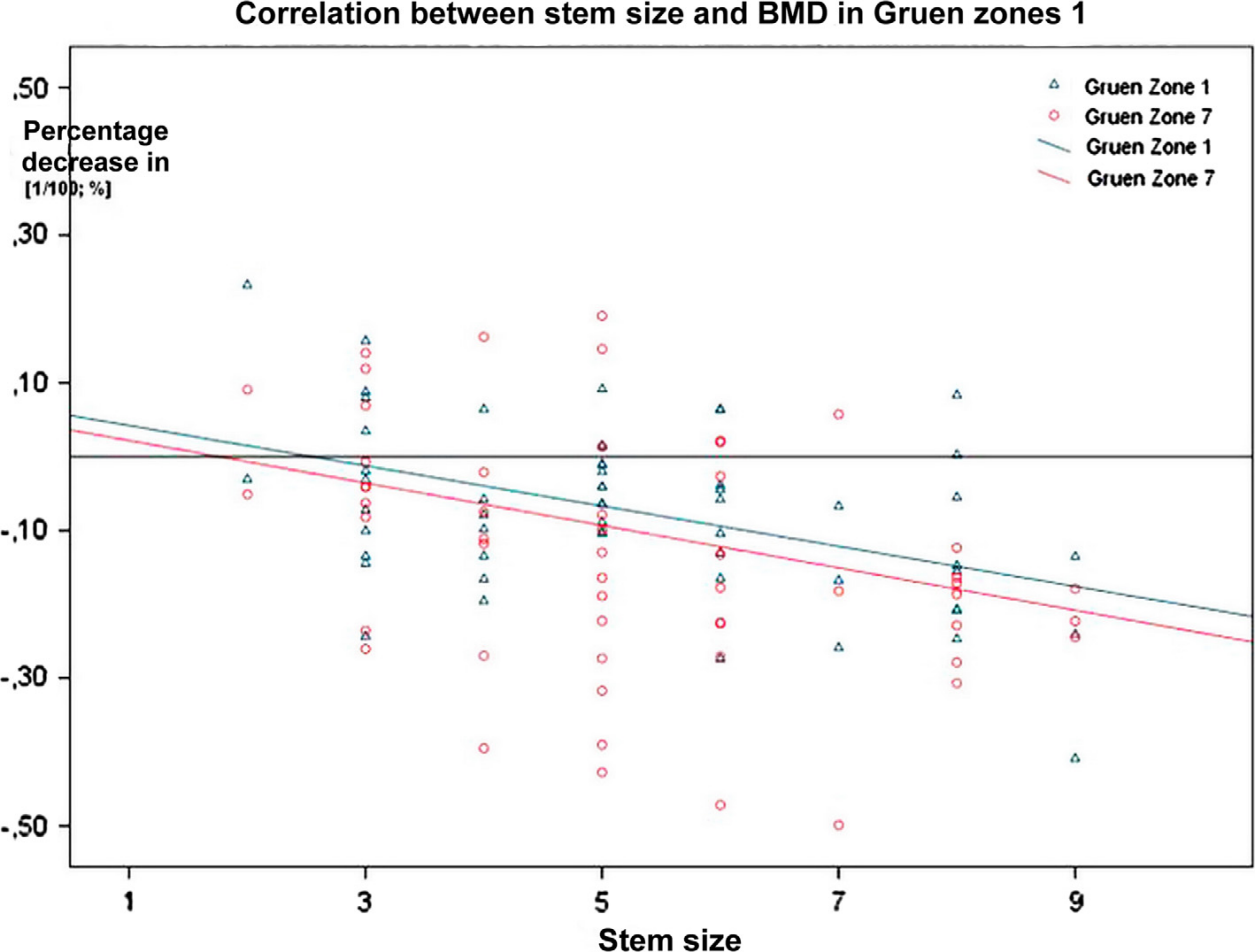
Correlation between stem size and bone density change after one year.

Correlation analysis depicts a weak correlation between the stem size and the BMD change in Gruen zone 7 (*r* = −0.213) without significance. A low-to-medium correlation as shown in Gruen zone 1 with significant correlation coefficient (*r* = −0.305) (Figure 5) was noticed.

**Figure 6.**
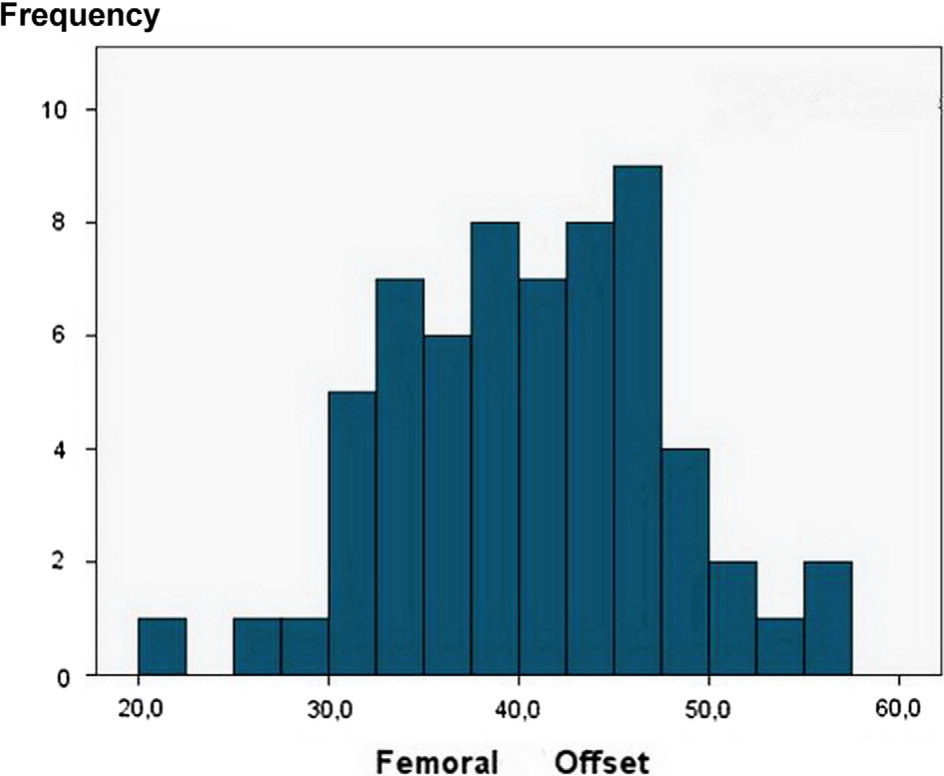
Distribution histogram showing femoral offset in (mm).

The average femoral offset was 40.5 mm. The frequency of femoral offsets among patients is shown in Figure 6. The correlation analysis showed no significance.

**Figure 7.**
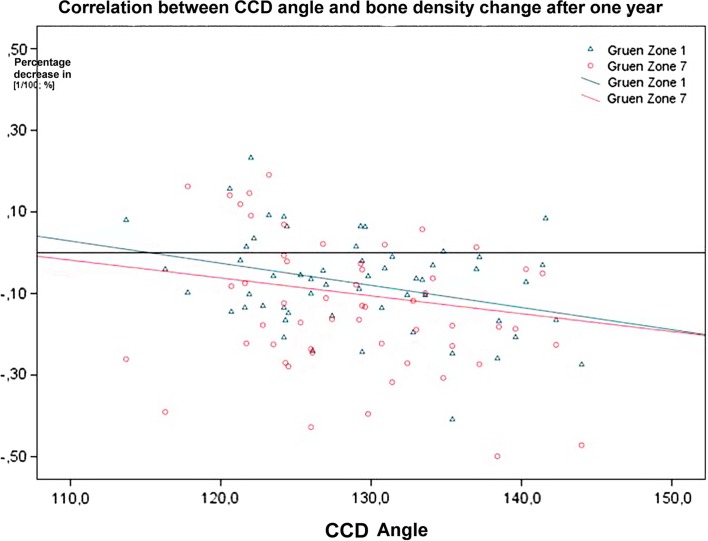
Correlation between CCD angle and bone density change after one year.

The average projected CCD angle after the implantation of MiniHip^TM^ was 128.8°. The correlation analysis showed a correlation coefficient *r* of −0.333. However, weak significant correlation between postoperative CCD angle and the bone density change in Gruen zone 1 after one year was observed. Also weak correlation was found for Gruen zone 7 (*r* = −0.131) (Figure 7).

The mean *T*-score at the lumbar spine among patients was 0.005. The correlation analysis showed no significance either for Gruen zone 1 or for Gruen zone 7.

In order to find out more predictors a covariance for all Gruen zones was carried out separately. The following variables were tested: Gender, side, preoperative diagnoses, CCD subgroups as well as subgroup pairings (Table 5). None of the above variables showed a significant difference in bone loss in the individual subgroups in the Gruen zones 1–7.

**Table 5.** Analysis of covariance.

Variable		Dataset with missing values	Pooled dataset
Gender	Female	*n* = 20	*n* = 34
	Male	*n* = 13	*n* = 28
Side	Left	*n* = 16	*n* = 31
	Right	*n* = 17	*n* = 31
Diagnosis	Osteoarthritis	*n* = 28	*n* = 49
	Hip dysplasia	*n* = 4	*n* = 7
	Avascular necrosis	*n* = 1	*n* = 4
	Femoral neck fracture	*n* = 0	*n* = 2
CCD angle	Varus < 120°	*n* = 1	*n* = 5
	Physiological 120°–135°	*n* = 25	*n* = 44
	Valgus > 135°	*n* = 7	*n* = 13
Friction pairings	Ceramic-ceramic	*n* = 20	*n* = 42
	Mixture	*n* = 13	*n* = 20

## Discussion

The most noteworthy finding in the current study was that periprosthetic bone resorption is a multifactorial process where stem size, CCD angle, and patient-specific variables such as the *T*-score have impact on the periprosthetic bone remodeling. In particular, this applies to Gruen zone 1.

To assess the change in bone density around the implanted prosthesis, the so-called DEXA scan has gained acceptance in recent years and decades. With this method it is possible to observe the reaction of the periprosthetic bone to the prosthesis implantation more accurately. DEXA scan has several advantages, particularly in terms of precision, reproducibility as well as radiation exposure. Previous studies showed an in vivo measurement repetition error of 2–3% [15, 16]. The DEXA scan used in the current study has measurement inaccuracies at below 2% [16, 17]. With DEXA scan, smaller bone density differences (4–5%) could be quantifiable, while this is only possible for bone density differences more than 30% when using conventional X-rays [15, 16].

Depending on the rotation of the thigh, region-dependent differences of about 10% could be found while only the proximal Gruen zones are prone to errors in different rotational positions. Studies with preoperative comparative measurements, such as control measurements at healthy hips, showed a bone density variance of up to 20% depending on the region [16]. Thus a study design with the other side as a comparison parameter is unsuitable.

Studies with measurements comparing preoperative and postoperative BMD showed density increases from an average of 7.7% to 9.0% in Gruen zones independently, although the stem preparation was performed with a rasp. BMD region dependent measurements preoperatively and postoperatively in the same patient showed 10–24% increase, especially in the proximal Gruen zones with the maximum in Gruen zone 1.

As in the first weeks after prosthesis implantation, the greatest change occurs, the reference measurement should be performed within two weeks after surgery [18]. For observation of the periprosthetic BMD changes, the investigation period of the first twelve months appeared to make the most sense, this is because it was demonstrated that in the first year postoperatively the most dynamic change in the BMD occurs [19]. The existing DEXA studies and Finite Element Analysis in particular for short stem prosthesis show that the largest changes take place in the first three to six months after surgery and after one year a plateau is reached. In the following one to two years slow changes occurred [20, 21]. Accordingly, this study was conducted.

The MiniHip^TM^ shows in the first three months a globally strong proximal bone density reduction. To some extent, the bone density decreases postoperatively in another three to six months, afterwards the periprosthetic bone density consolidates. A virtually “steady-state” (state of equilibrium) is achieved. The global periprosthetic bone loss in the first three to six months postoperatively after the implantation of uncemented short shaft prothesis in the current study coincides with the results of other studies [17, 19, 21–24].

For the initial loss of bone density after implantation of the MiniHip^TM^ femoral neck-sustaining short stem prosthesis, the compression of the periprosthetic cancellous bone intraoperatively plays a significant role. Also other cementless hip implants, in which the femoral medullary cavity is opened with a rasp or impactor, show global atrophy in the first few months as well. This is a result of bone redistribution [17, 19, 23–25]. In the MiniHip^TM^, no rasp is used as in standard hip replacements, but a medullary compressor, so, here bone redistribution can be expected even more. In addition, mechanical manipulation during surgery may lead to differences in bone density when comparing preoperatively and postoperatively by more than 20% [21].

Whether the pressures resulting from impaction are so high that bone formation is induced or suppressed was always a valid question. Gruen was able to show that the impaction of periprosthetic cancellous with resultant primary stability improves and reduces micro-movements [26]. Another significant factor is the postoperative full weight bearing of the patients, which also contributed at least a part to the bone density reduction [17].

Load transmission changes after prosthesis implantation is another factor which leads to decrease bone density because of “stress shielding” [27].

The moderate bone loss after one year in the proximal Gruen regions is also confirmed by studies of other neck partially-sustaining short stems such as Metha short stem prosthesis [21, 28]. Good results were also shown using the Nanos short stem prosthesis [25]. From the group of femoral neck partially-sustaining short shaft prostheses systems the CFP stem showed bone loss of more than 30% in the Gruen zone 7 [24]. In the group of femoral neck resected short stem systems, the Mayo prosthesis shows slightly larger losses in bone density after one year in both proximal Gruen zones [23]. The proxima, however, shows a positive bone turnover in both Gruen zones after one year [29].

Bone density studies of conventional prostheses [straight-stemmed CLS Spotorno (Fa. Zimmer), ABG 1 or 2 (Fa. Stryker)] a significantly higher bone loss especially in Gruen zone 7 than the MiniHip^TM^ [19, 22, 30]. One explanation for the behavior of the standard shaft prostheses is that the stress shielding increases by the increase in stem length and thus the proximal force transmission is reduced [31, 32].

The conventional prostheses systems have in common the fact that the Gruen Zone 1 is less affected by bone demineralization. This is partly explained by the tensile forces of the gluteal muscles [33]. The lower distal bone density reduction in short stem prostheses is probably the basis for why thigh pain is having a lower incidence [34].

Literature indicates that significant bone density loss is not related sometimes to clinical results. Both the cemented Spotorno^®^ and the cementless Bicontact^®^ Stem showed excellent long-term clinical results, compared to cemented prostheses [22, 35]. The long-term study conducted by Aldinger et al. [22] with the Spotorno^®^ stem with seven-year DEXA scan showed results comparable to those of the current study.

The fate of a cementless short stem prosthesis implanted depends essentially on the processes at the bone-implant interface. In the course of this, one of the most important requirements of osteogenic competence is an impeccable primary stability [6].

Another influential factor seems to have a role in the current study which is the stem size, having a significant negative correlation to bone changes in the Gruen zone 1. In Gruen zone 7, the same tendency is at least recognizable. While some studies were also able to demonstrate this relationship [36–38], others could not establish connection between stem size and bone loss [20, 22].

Correlation analysis between femoral offset and BMD change showed no significant relationship. The restoration of the femoral offset is important for the joint stability and the durability of the replacement hip joint [14].

In Gruen zone 7, the same negative trend of high CCD angle was recognizable with higher probability of bone resorption. These relationships could be explained as follows: at high CCD angles and resection plane roughly equivalent to the standard resection of conventional shafts and in the presence of stress shielding forces and periprosthetic bone remodeling that take place in the same time, this allows for higher proximal bone density loss. Dividing bone density decrease depending on the CCD into three groups (valgus hip > 135°, physiological hip 120–135°, varus hips < 120°) in the current study and comparing them with each other, there were no significant differences among the three groups. In the literature, there are conflicting results concerning this. Panisello showed in his study that both varus as well as valgus positionings led to a significant decrease in BMD mainly in Gruen zone 7 [39].

Strong points of the current study are: (1) To our knowledge, this is the first record of periprosthetic bone density changes after MiniHip^TM^ cementless femoral short stem. (2) Measurements were performed within two weeks postoperatively, three, six, and twelve months postoperatively as recommended by the literature. Weak points are: (1) No comparison between periprosthetic bone density changes after MiniHip^TM^ and after other short stem prostheses. (2) No comparison between periprosthetic bone density changes after MiniHip^TM^ and after other conventional prostheses. (3) Vit.-D levels were not measured. (4) Osteoporosis medications were not assessed. (5) Correlation between the increase in bone density and clinical results in general was not performed.

## Conclusions

MiniHip^TM^ densitometric results are promising and are comparable to good results of the other representatives of the femoral neck partially-sustaining short stem prostheses with a lower proximal bone density reduction.

Periprosthetic bone resorption is a multifactorial process where stem size, and CCD angle and patient-specific variables such as the *T*-value have an impact on the periprosthetic bone remodeling. In particular, this applies to the Gruen zone 1.

## Conflict of interest

The authors declare that they have no conflict of interest. Also, they haven't received any payment or services from any third party for any aspect of the submitted work. Also, they have no financial relationships with any the entities described in the instructions. Also, the authors have no patents whether planned, pending or issued
